# AI Applied to Formulation Design, Process Optimization, and Quality Control in 3D Printed Drug Products: Protocol for a Scoping Review

**DOI:** 10.2196/98251

**Published:** 2026-08-18

**Authors:** Patricia Garcia Ferreira, Caroline Deckmann Nicoletti, Vitor Francisco Ferreira, Tácio de Mendonça Lima

**Affiliations:** 1 Department of Pharmacy and Pharmaceutical Administration Universidade Federal Fluminense Niterói, Rio de Janeiro Brazil

**Keywords:** 3D printing, additive manufacturing, artificial intelligence, AI, formulation design, process optimization

## Abstract

**Background:**

3D printing is an advanced, computer-aided design (CAD)–guided, layer-by-layer manufacturing technology with strong potential for producing patient-specific drug products. Integrating AI with pharmaceutical 3D printing can accelerate formulation development, improve process robustness and quality, and enable efficient personalization across the preprinting, printing, and postprinting stages. However, evidence remains dispersed across technologies, dosage forms, and AI approaches, making it difficult to obtain a comprehensive understanding of current research and knowledge gaps.

**Objective:**

This scoping review protocol aims to map and characterize how AI has been applied to the formulation design, process optimization, and quality control of 3D printed drug products across the preprinting, printing, and postprinting stages.

**Methods:**

This review will follow the Joanna Briggs Institute (JBI) methodology and the PRISMA-ScR (Preferred Reporting Items for Systematic Reviews and Meta-Analyses Extension for Scoping Reviews) checklist and guidelines for scoping reviews. The following databases and resources will be searched: MEDLINE (PubMed), Scopus, and Web of Science. Screening, data extraction, and data analysis will be conducted by 2 reviewers independently. Findings will be presented in summary tables and a narrative synthesis.

**Results:**

The protocol and search strategy were finalized in April 2026, and the review was registered on the Open Science Framework (OSF). Literature searches are scheduled to be conducted between May 2026 and June 2026, followed by title and abstract screening and full-text assessment between June 2026 and August 2026. Data extraction and narrative synthesis are expected to be completed by September 2026. The final scoping review is expected to be submitted for publication in the fourth quarter of 2026.

**Conclusions:**

This protocol outlines a scoping review to map the use of AI in the formulation design, process optimization, and quality control of 3D printed drug products, and summarize current research and identify areas for future investigation.

**Trial Registration:**

Open Science Framework 10.17605/OSF.IO/2R8ED; https://osf.io/2r8ed/overview

**International Registered Report Identifier (IRRID):**

PRR1-10.2196/98251

## Introduction

Contemporary medicine is undergoing profound changes driven by the advancement of digital technologies and novel manufacturing approaches, with 3D printing playing a prominent role in this transformation. 3D printing has established itself as an advanced rapid-prototyping technology that enables the layer-by-layer fabrication of complex objects under the precise guidance of computer-aided design (CAD) models. Its distinctive manufacturing paradigm offers multiple advantages and is particularly suited to the production of personalized, complex structures, with substantive progress reported across diverse fields [1]. In pharmaceutics, 3D printing holds revolutionary potential for producing patient-specific drug products, offering broad flexibility in dose, structure, and dosage-form size to meet individual needs. This capability enables the manufacture of precision and personalized medicines tailored to individual patients, with the potential to improve therapeutic efficacy and clinical outcomes. Notwithstanding this promise, pharmaceutical 3D printing remains at an early stage of development [2].

Given that 3D printing is intrinsically digital and aligned with personalized therapy, it is prudent for the pharmaceutical sector to integrate state-of-the-art methods with AI-enabled 3D printing. To accelerate progress rapidly and sustainably, in silico tools are increasingly being adopted, and AI has emerged as a transformative approach with growing potential to enhance pharmaceutical 3D printing technologies [1].

The combination of these technologies not only improves the quality of 3D-printed pharmaceutical products but also promotes cost efficiency, accelerates production, and reduces waste, thereby perfectly aligning with the principles of the circular economy. Such integration could advance the industry and fully capitalize on current technological capabilities. Moreover, the large volumes of data generated by ongoing research in pharmaceutical 3D printing can be leveraged by machine learning techniques to refine formulations and predict outcomes, encompassing formulation design (before printing), process optimization (during printing), and quality control (after printing) [3-6].

In the preprinting phase, AI supports the selection and proportioning of excipients (polymers, plasticizers, solvents, and drug load), rheological characterization, and the definition of product targets (eg, release profile, mechanical strength, and disintegration), thereby reducing trial-and-error experimentation and anticipating expected performance while saving time and resources. During the printing phase, AI assists in tuning parameters, such as pressure or force, extrusion speed, travel speed, layer height, temperature, and infill pattern, to optimize dimensional accuracy, process robustness, and efficiency. Consequently, it accelerates development, improves quality, and enables personalization more efficiently [7-9]. In the postprinting phase, AI enables real-time monitoring of pharmaceutical manufacturing processes and the control of prespecified critical quality attributes (CQAs) and critical process parameters (CPPs) [10]. Accordingly, this scoping review aims to systematically map and characterize the available evidence on the application of AI to formulation design, process optimization, and quality control of 3D printed drug products across the preprinting, printing, and postprinting stages. The review will identify the types of evidence available, the pharmaceutical 3D printing technologies investigated, the AI approaches used, and the targeted CQAs and CPPs reported in the literature.

## Methods

### Design

This scoping review will be conducted in accordance with the Joanna Briggs Institute (JBI) Manual for Evidence Synthesis and reported following the PRISMA-ScR (Preferred Reporting Items for Systematic Reviews and Meta-Analyses Extension for Scoping Reviews) checklist and guidelines [11,12]. The review will address the following questions:

- What evidence is available regarding the application of AI in the formulation design of 3D printed drug products?
- What AI approaches have been reported for process optimization in pharmaceutical 3D printing?
- What evidence is available regarding the use of AI for quality control and postprinting monitoring of 3D printed drug products?
- How are AI methods, pharmaceutical dosage forms, and 3D printing technologies represented across the current literature?

The review protocol will be registered on the Open Science Framework (OSF) platform, and any deviations will be justified in the final report.

### Eligibility Criteria

#### Participants

This scoping review will consider studies involving pharmaceutical drug products and therapeutic systems manufactured through 3D printing technologies and containing one or more active pharmaceutical ingredients (APIs). Eligible studies may include conventional pharmaceutical dosage forms, such as tablets, capsules, films, gels, implants, and other drug-loaded therapeutic systems designed for drug delivery or therapeutic purposes. Medical devices, prostheses, and personalized therapeutic systems will be considered only when they incorporate APIs and when AI is applied to formulation design, process optimization, or quality control.

Studies involving biomedical devices without APIs, tissue-engineering constructs, bioprinted tissues, regenerative medicine applications, and nonpharmaceutical medical devices will be excluded.

Studies using different pharmaceutical 3D printing technologies will be considered, including semisolid extrusion (SSE), fused deposition modeling (FDM), stereolithography or digital light processing (DLP), selective laser sintering (SLS), and inkjet or binder jetting technologies [13-16].

#### Concept

The concept of interest is the application of AI to formulation design, process optimization, and quality control in pharmaceutical 3D printing. AI approaches may include machine learning, deep learning, computer vision, generative models, Bayesian optimization, and related computational methods trained using experimental, formulation, rheological, imaging, or process-related datasets [4-10,17].

The review will consider AI applications across the preprinting, printing, and postprinting stages of pharmaceutical additive manufacturing. These applications may include formulation prediction, printability assessment, optimization of process parameters, monitoring of CPPs, prediction or evaluation of CQAs, and postprinting quality control strategies [6-14,17].

#### Context

There will be no restrictions regarding the setting or geographic location.

### Types of Sources

The proposed scoping review will consider primary study designs, such as developmental studies.

Studies that do not clearly describe the use of AI in pharmaceutical 3D printing technologies will be excluded. Additionally, literature reviews, letters, editorials, comments, books or book chapters, guidelines, conference abstracts or proceedings, and studies without full-text availability will be excluded.

### Search Strategy and Databases

The search strategy will be guided by the PRISMA statement for reporting literature searches in systematic reviews (PRISMA-S [Preferred Reporting Items for Systematic Reviews and Meta-Analyses Literature Search Extension]) [18]. A 3-step search strategy will be used to identify studies and sources. First, an initial search of the PubMed database will be performed to identify articles on the topic. The controlled vocabulary of the National Library of Medicine, through the MeSH, along with keywords and terms related to 3D printed drug products and AI contained in the relevant articles, will be used to develop a full search strategy. The full search strategy will be adapted for each database, with consideration of its specific indexed terms. The reference lists of all included sources of evidence will be screened for additional studies. No restrictions will be applied regarding language or publication year. The literature search will be conducted in the MEDLINE (PubMed), Scopus, and Web of Science databases. Textbox 1 outlines the search strategy and databases.

**Search strategies used for each database.**

**MEDLINE (PubMed; searched on May 15, 2026; 206 records retrieved; no restrictions on language or publication year)**

1. “3D print*”[tiab] OR “Three-Dimensional Print*”[tiab] OR “Additive Manufacturing”[tiab] OR “3D Printed Formulation”[tiab] OR “3D Printed Drug*”[tiab] OR “3D Printed Medicine”[tiab] OR “Printing, Three-Dimensional”[MeSH]
2. “Artificial Intelligence”[tiab] OR “Machine Learning”[tiab] OR “AI”[tiab] OR “Neural Networks”[tiab] OR “Deep Learning”[tiab] OR “Generative AI”[tiab] OR “Artificial Intelligence”[MeSH] OR “Machine Learning”[MeSH]
3. “Personalized Pharmaceuticals”[tiab] OR “Pharmaceutical Preparation”[tiab] OR “Pharmaceutical Product”[tiab] OR “Pharmaceutical Manufacturing”[tiab] OR “Drug Delivery*”[tiab] OR “Drug Development”[tiab] OR “Personalized Medicines”[tiab] OR “Innovative Drug Formulations”[tiab] OR “Pharmaceutical Preparations”[MeSH] OR “Drug Delivery Systems”[MeSH]
4. #1 AND #2 AND #3 

**Scopus (searched on May 15, 2026; 199 records retrieved; no restrictions on language or publication year)**

TITLE-ABS-KEY((“3D Print*” OR “Three-Dimensional Print*”OR “Additive Manufacturing” OR “3D Printed Formulation” OR “3D Printed Drug*” OR “3D Printed Medicine” OR “Printing, Three-Dimensional”) AND(“Artificial Intelligence” OR “Machine Learning” OR “AI” OR “Neural Networks” OR “Deep Learning” OR “Generative AI” OR “Artificial Intelligence” OR “Machine Learning”) AND (“Personalized Pharmaceuticals” OR “Pharmaceutical Preparation” OR “Pharmaceutical Product” OR “Pharmaceutical Manufacturing” OR “Drug Delivery*” OR “Drug Development” OR “Personalized Medicines” OR “Innovative Drug Formulations” OR “Pharmaceutical Preparations” OR “Drug Delivery Systems”)) AND NOT (DOCTYPE(“re” OR “cp” OR “er” OR “cr” OR “bk” OR “ch” OR “sh”) OR SRCTYPE(“b” OR “k” OR “p” OR “n” OR “w” OR “l”))

**Web of Science (searched on May 15, 2026; 240 records retrieved; no restrictions on language or publication year)**

1. TS=(“3D Print*” OR “Three-Dimensional Print*” OR “Additive Manufacturing” OR “3D Printed Formulation” OR “3D Printed Drug*” OR “3D Printed Medicine” OR “Printing, Three-Dimensional”)
2. TS=(“Artificial Intelligence” OR “Machine Learning” OR “AI” OR “Neural Network*” OR “Deep Learning” OR “Generative AI”)
3. TS=(“Pharmaceutical Preparation*” OR “Pharmaceutical Product*” OR “Pharmaceutical Manufacturing” OR “Drug Delivery*” OR “Drug Development” OR “Personalized Medicine” OR “Personalized Pharmaceutical*”)
4. #1 AND #2 AND #3

### Source of Evidence Selection

Studies retrieved from the databases will be imported into the Rayyan (Rayyan Systems Inc) platform for duplicate removal [19]. The titles and abstracts will be screened by 2 reviewers independently (PF and CDN) to identify potentially eligible studies. Full texts of relevant articles will be assessed for final inclusion based on predefined eligibility criteria. Disagreements will be resolved by consensus or through consultation with a third reviewer (TDML). If full-text access is unavailable, corresponding authors will be contacted via email and/or the ResearchGate platform. Sources that fail to meet the eligibility criteria will be excluded, and the reasons for exclusion will be reported in the scoping review.

### Data Extraction

Two independent reviewers (PF and CDN) will perform data extraction and charting using a predeveloped and pilot-tested Microsoft Excel form. Disagreements will be resolved through discussion or by consultation with a third reviewer (TDML). The charting process will be iterative, allowing the refinement of categories and variables throughout the review process, in accordance with JBI recommendations for scoping reviews. The extracted data will include bibliographic information (author, year, country, and study type), characteristics of the pharmaceutical 3D printed products and APIs, 3D printing technologies, AI methods and tasks, data inputs, targeted critical material attributes (CMAs) and CPPs, CQAs, quality control strategies, validation approaches, and reported process performance indicators (PPIs). The charted information will also include details regarding the stage of pharmaceutical additive manufacturing at which AI was applied (preprinting, printing, or postprinting stage), the intended pharmaceutical application, and the reported methodological approaches used for optimization, prediction, monitoring, or quality assessment. If necessary, corresponding authors of included studies will be contacted to obtain missing or additional information.

### Data Analysis and Presentation

The extracted evidence will be synthesized descriptively and presented through tabular, graphical, and narrative approaches. The synthesis will focus on mapping the extent, characteristics, and distribution of the available evidence regarding the application of AI in pharmaceutical 3D printing. Studies will be grouped according to key characteristics, including pharmaceutical dosage forms, 3D printing technologies, AI methods, targeted CQAs and CPPs, and stages of additive manufacturing (preprinting, printing, and postprinting stages). The review will also summarize the reported purposes of AI applications, such as formulation prediction, printability assessment, process optimization, process monitoring, and quality control. Summary tables will be developed to organize study characteristics, methodological approaches, AI tasks, validation strategies, and reported performance metrics. A narrative synthesis will accompany the tabulated results to describe patterns, methodological trends, research gaps, and inconsistencies across the literature. Additionally, a Sankey diagram will be constructed to visually represent the co-occurrence of pharmaceutical 3D printing technologies and AI approaches identified in the included studies.

## Results

The protocol, eligibility criteria, and search strategy were finalized in April 2026, and the review was registered on the OSF platform. Database searches are planned for May and June 2026, with study selection and full-text screening expected to occur between June and August 2026. Data extraction, synthesis, and the development of the Sankey diagram are projected to be completed by September 2026. Submission of the final scoping review manuscript is anticipated in the fourth quarter of 2026.

## Discussion

### Principal Findings

This scoping review aims to provide a comprehensive mapping of how AI has been incorporated into pharmaceutical 3D printing workflows, including formulation design, process optimization, and quality control of drug products manufactured through additive manufacturing technologies. On the basis of preliminary searches, we anticipate that the available evidence will be concentrated on a limited number of pharmaceutical 3D printing platforms, particularly FDM, SSE, and SLS, while other technologies remain comparatively underexplored.

We also expect substantial heterogeneity in AI methodologies, datasets, validation strategies, and reported performance metrics. Machine learning algorithms, deep learning models, computer vision systems, and optimization approaches are likely to represent the predominant AI categories identified. However, the extent to which these methods are externally validated and transferable to real-world pharmaceutical manufacturing environments remains unclear. Therefore, this review is expected to identify important methodological trends, evidence gaps, and opportunities for standardization across the emerging field of AI-assisted pharmaceutical 3D printing.

Furthermore, by organizing evidence according to the stages of additive manufacturing (preprinting, printing, and postprinting stages), dosage form, AI task, and targeted CQAs, this review will provide a structured overview of how AI is currently being used to support quality by design principles and data-driven pharmaceutical development.

### Comparison to Prior Work

Several reviews have independently explored pharmaceutical 3D printing, AI in drug development, and digital transformation in pharmaceutical manufacturing [19-24]. Previous publications have demonstrated the growing potential of additive manufacturing for personalized medicines and highlighted the increasing use of machine learning to support formulation development and process optimization. However, most available reviews focus either on 3D printing technologies or on AI applications in pharmaceutical sciences more broadly, without specifically examining the intersection of these rapidly evolving fields [25-30].

To our knowledge, no previous scoping review has systematically mapped the application of AI across the entire pharmaceutical 3D printing workflow, encompassing formulation design, printability prediction, process optimization, process monitoring, and postprinting quality control. Existing reports typically describe isolated case studies, specific machine learning models, or individual manufacturing technologies, making it difficult to obtain an integrated understanding of current research trends.

### Strengths and Limitations

This protocol presents several important strengths. First, the review will be conducted according to the methodological guidance provided by the JBI and reported following the PRISMA-ScR framework, ensuring methodological transparency and reproducibility. Second, a comprehensive search strategy will be applied across multiple databases without restrictions regarding publication year or language, maximizing the likelihood of identifying relevant evidence. Third, the review will capture a broad range of pharmaceutical applications, additive manufacturing technologies, AI approaches, and quality-related outcomes, allowing a comprehensive mapping of the field.

An additional strength is the planned extraction of detailed methodological information related to AI development and validation, including model type, training and testing strategies, validation approaches, performance metrics, and data availability. This information may help identify recurring methodological weaknesses and reporting deficiencies that could affect reproducibility and generalizability.

Nevertheless, several limitations should be acknowledged. As a scoping review, the objective is to map and characterize the available evidence rather than evaluate effectiveness or establish causal relationships. Consequently, no formal risk-of-bias assessment will be performed. In addition, the review may be affected by substantial heterogeneity in study designs, additive manufacturing technologies, datasets, AI architectures, and outcome reporting. The rapidly evolving nature of both pharmaceutical 3D printing and AI may also result in publication lag, meaning that some emerging technologies may not yet be adequately represented in the literature. Finally, variability in validation practices and the limited availability of external validation datasets may restrict the interpretation of reported model performance.

### Future Directions

The findings of this review may help define priorities for future research at the interface of AI and pharmaceutical additive manufacturing. Future investigations should emphasize the development of standardized reporting frameworks, harmonized performance metrics, and robust validation methodologies to improve reproducibility and facilitate comparison across studies.

Particular attention should be directed toward external validation, multicenter datasets, explainable AI approaches, and regulatory-ready methodologies capable of supporting pharmaceutical quality assurance. Additionally, future research should explore the application of advanced AI techniques, including generative AI, reinforcement learning, multimodal learning, and autonomous optimization systems capable of supporting inverse formulation design and adaptive manufacturing processes. These developments may accelerate the transition toward fully digitalized and personalized pharmaceutical production systems aligned with the principles of Pharma 4.0.

### Dissemination Plan

The findings of this scoping review will be disseminated through publication in a peer-reviewed scientific journal and presentation at national and international conferences related to pharmaceutical sciences, pharmaceutical technology, additive manufacturing, and AI. By providing a comprehensive overview of current evidence and knowledge gaps, this review aims to support future research initiatives, foster interdisciplinary collaboration, and contribute to the development of evidence-based strategies for integrating AI into pharmaceutical 3D printing workflows. The findings may also inform regulatory discussions and facilitate the adoption of data-driven approaches in personalized medicine and advanced pharmaceutical manufacturing.

### Conclusions

This protocol describes the methodology for a scoping review that will map the application of AI to formulation design, process optimization, and quality control of 3D printed drug products. By systematically identifying and characterizing the available evidence, the review aims to provide an overview of current research activity, highlight areas requiring further investigation, and support future research and development at the intersection of AI and pharmaceutical additive manufacturing.

## Abbreviations

API: active pharmaceutical ingredient
CAD: computer-aided design
CMA: critical material attribute
CPP: critical process parameter
CQA: critical quality attribute
DLP: digital light processing
FDM: fused deposition modeling
JBI: Joanna Briggs Institute
OSF: Open Science Framework
PPI: process performance indicator
PRISMA-S: Preferred Reporting Items for Systematic Reviews and Meta-Analyses Literature Search Extension
PRISMA-ScR: Preferred Reporting Items for Systematic Reviews and Meta-Analyses Extension for Scoping Reviews
SLS: selective laser sintering
SSE: semisolid extrusion
